# Integration of Human African Trypanosomiasis Control Activities into Primary Health Services in the Democratic Republic of the Congo: A Qualitative Study of Stakeholder Perceptions

**DOI:** 10.4269/ajtmh.18-0382

**Published:** 2019-02-04

**Authors:** Philippe Mulenga, Marleen Boelaert, Pascal Lutumba, Catiane Vander Kelen, Yves Coppieters, Faustin Chenge, Crispin Lumbala, Oscar Luboya, Alain Mpanya

**Affiliations:** 1 Faculty of Medicine and School of Public Health, University of Lubumbashi, Lubumbashi, DRC;; 2 Department of Public Health, Institute of Tropical Medicine, Antwerp, Belgium;; 3 School of Public Health, Université Libre de Bruxelles, Brussels, Belgium;; 4 Department of Tropical Medicine, Faculty of Medicine, University of Kinshasa, Kinshasa, DRC;; 5 National Program for the Control of Human African Trypanosomiasis, Kinshasa, DRC

## Abstract

Human African trypanosomiasis is close to elimination in several countries in sub-Saharan Africa. The diagnosis and treatment is currently rapidly being integrated into first-line health services. We aimed to document the perspective of stakeholders on this integration process. We conducted 12 focus groups with communities in three health zones of the Democratic Republic of the Congo and held 32 interviews with health-care providers, managers, policy makers, and public health experts. The topic guide focused on enabling and blocking factors related to the integrated diagnosis and treatment approach. The data were analyzed with NVivo (QSR International, Melbourne, Australia) using a thematic analysis process. The results showed that the community mostly welcomed integrated care for diagnosis and treatment of sleeping sickness, as they value the proximity of first-line health services, but feared possible financial barriers. Health-care professionals thought integration contributed to the elimination goal but identified several implementation challenges, such as the lack of skills, equipment, motivation and financial resources in these basic health services. Patients often use multiple therapeutic itineraries that do not necessarily lead them to health centers where screening is available. Financial barriers are important, as health care is not free in first-line health centers, in contrast to the population screening campaigns. Communities and providers signal several challenges regarding the integration process. To succeed, the required training of health professionals, as well as staff deployment and remuneration policy and the financial barriers in the primary care system need to be addressed, to ensure coverage for those most in need.

## INTRODUCTION

Human African trypanosomiasis (HAT) or “*sleeping sickness*” is a vector-borne parasitic disease that mainly affects poor people living in rural areas of sub-Saharan Africa. Two species of parasites can cause sleeping sickness, *Trypanosoma brucei gambiense* and *T. brucei rhodesiense*.^1–3^ The form caused by *T. brucei gambiense* is anthroponotic and is found in 24 countries of sub-Saharan Africa, including the Democratic Republic of the Congo (DRC) that accounts for a large part of the current disease burden. The *gambiense* form causes 98% of the global cases of HAT.^4^ The disease progresses in two stages, the first or hemolymphatic stage and the second or meningoencephalitic stage. In the hemolymphatic stage, there are few clinical signs. When patients seek health care, they are usually already in the meningoencephalitic stage characterized by neurological and psychiatric signs.^5–7^ Human African trypanosomiasis control is usually based on two strategies: active case finding by mobile units followed by treatment in dedicated centers and vector control.^8–12^ The WHO is targeting the elimination of *T. brucei gambiense* HAT by 2020 and the interruption of its transmission to humans by 2030.^3,13,14^ The number of worldwide cases has diminished very drastically over the past years, from 26,500 cases reported in 2000 to 2,184 cases in 2016, of which 80% occur in the DRC.

Since 1968, the National Program for the Control of HAT (PNLTHA) in the DRC has set up dedicated HAT diagnosis and treatment centers, entirely managed by the program. In addition, PNLTHA has been integrating certain HAT control activities into a number of general hospitals and first-line health centers (PNLTHA-DRC, unpublished data). In these primary care centers, the “integrated HAT care” is mostly limited to the screening step in the diagnostic process, with only a few upgraded centers offering also confirmation and treatment.

With the steady decline of the number of HAT cases globally and in the DRC, this integrated approach takes a major importance. Today more than half of the HAT cases are not detected by active case finding, but by fixed health facilities in the so-called “passive” mode. This integration process has been the subject of debate for many years. Authors have argued in favor of integration into basic health services for various reasons: excessive costs of mobile units in a context of drastic reduction in the number of cases^15^; inadequate services,^16^ lack of sustainability,^17^ and the negative resource impact of disease-specific (or “vertical”) control programs on the functioning of the health system (Ministry of Public Health-DRC, unpublished data). Others objected against the integration of a disease that has become so rare, into first-line health services that are not functioning very well. According to these authors, health workers hardly see any HAT cases to the point that their ability to manage these is difficult to maintain. Some authors recommend close supervision and a strict evaluation of integrated HAT control activities fearing loss of quality.^18^

The current status of the health services in DRC is reflecting the protracted socioeconomic crisis faced by the country. In 2015, Mitashi et al.^10^ drew a worrying picture of the state of the local health services in the Mushie and Kwamouth health districts in Bandundu, DRC, the epicenter of the HAT epidemic, based on observations of 43 first-level health centers. In general, these multipurpose health centers were located in poor buildings, they were poorly attended, there was a severe lack of equipment, and the technical competence of the laboratory staff was questionable. The authors concluded that basic health services in these two areas were not functional enough to integrate HAT control activities. Even relatively well-functioning health services may fail to detect HAT for several years because of the insidious spread of the disease or the fact that cases are living far away from the health centers.^19^

To achieve the goal of elimination, WHO recommends operational research on the integration of HAT into existing health services and on the optimization of passive screening, surveillance, and the management of these health services.^3^ We aimed to document the views of communities and health professionals in endemic areas and analyzed the factors that can positively or negatively influence the integration of HAT control in the local health services in DRC.

## METHODS

We conducted a qualitative study by focus group discussion (FGD) and in-depth interviews to understand the perceptions of stakeholders on the integration process of HAT control into primary health care (PHC) and to identify potential barriers.

**Figure 1.**
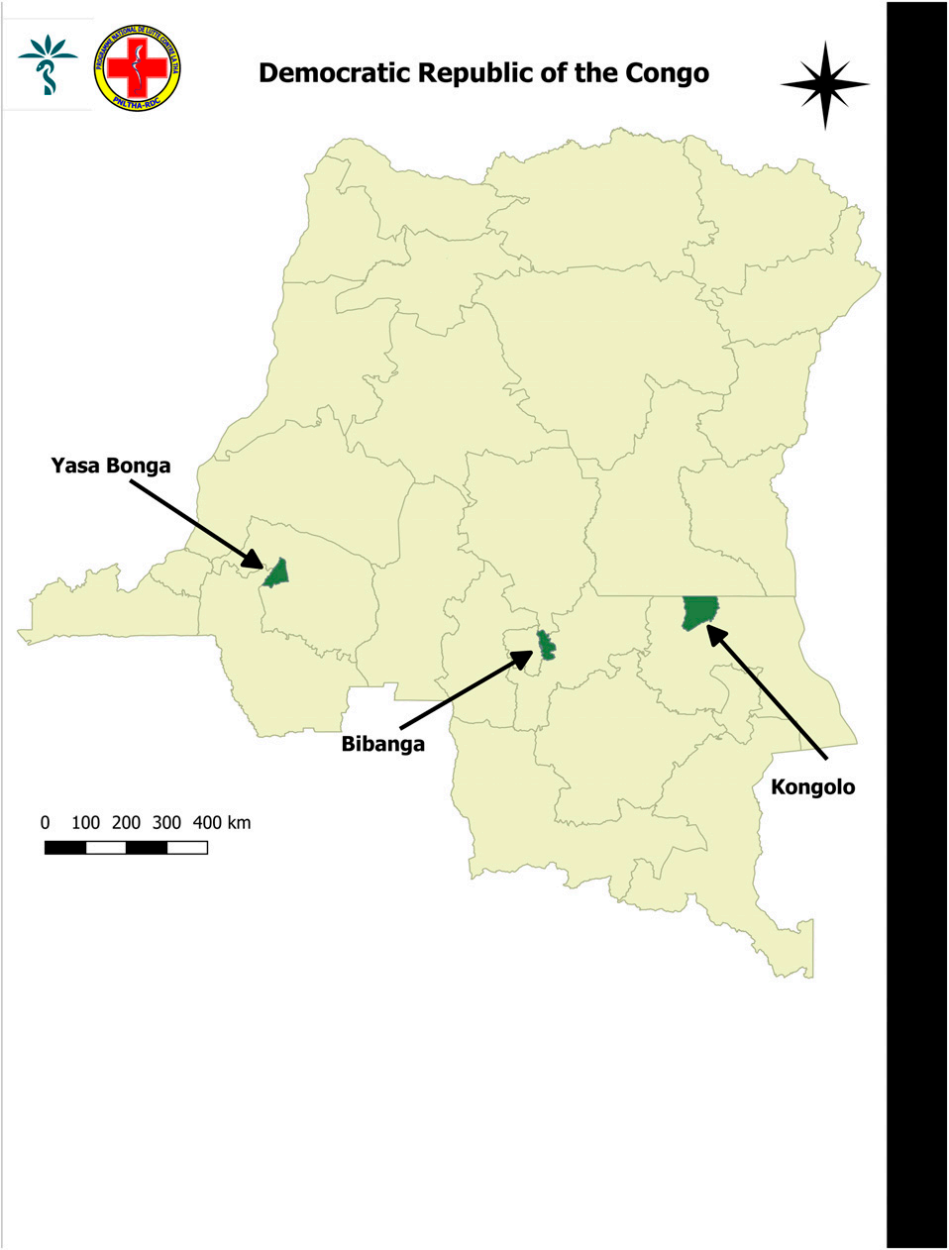
Map of Democratic Republic of the Congo with study sites “Health districts Yasa Bonga, Kongolo, and Bibanga (created using Quantum Geographic Information System 2.8 Las Palmas www.qgis.org).” This figure appears in color at www.ajtmh.org.

The study was conducted in three endemic areas of HAT. The health districts of Yasa Bonga, Bibanga, and Kongolo, which are located in the provinces of Kwilu, Kasaï Oriental, and Tanganyika in the DRC, respectively (Figure 1).

These three health zones were chosen according to the level of endemicity of the HAT (the Yasa Bonga and Bibanga zones have high and Kongolo has weak endemicity) and the level of functionality of the health zone (Kongolo and Bibanga work without and Yasa Bonga with financial support from external partners). These are two key factors that can positively or negatively influence the process of integrating HAT into PHC. In the following paragraphs, we report sampling, data collection and data analysis methods with community members and individual interviews with care providers, health system managers, and HAT and public health experts.

### Focus group discussions.

Between April and June 2015, 12 FGDs were carried out within the communities, that is four FGDs per health zone. These FGDs have been conducted to the point of saturation of information.^20^ Each FGD consisted of eight to 12 participants who were selected with the help of the village chiefs. We excluded people residing less than 2 years in the community and anyone who could exert undue influence on the responses of community members in the FGD process. The FGDs were composed homogenously by gender and by age group (under 35 years and older than 35 years) to allow participants to express themselves more freely. The FGDs took place in a quiet environment, e.g., inside an unoccupied building (school or church) or under a tree. The discussions were conducted in local languages (Kikongo, Tshiluba, and Kiswahili) and were recorded on a digital medium and lasted between 30 and 60 minutes. The FGDs were conducted by investigators who were previously trained in [facilitating FGDs as a] data collection method in social science. Our question guide included the following topics: perception of HAT and its control methods, barriers to HAT control, perceived need for HAT screening and treatment, opinions on the type and quality of service and community perspectives on HAT control.

### Individual interviews.

We conducted 32 interviews with key informants: six community leaders, six health-care providers, eight health system managers, eight public health professionals, and four researchers (see Table 1). The interviews lasted on average 30 minutes and were recorded on a digital medium. The principal investigator conducted the interviews in the language of the interviewee’s choice using an interview guide as support. This guide included the following themes: perceptions of integrating HAT into PHC, perceptions of the most appropriate interventions and type of service, the ideal way to integrate HAT activities into PHC, potential barriers, and factors that may contribute to the integration of HAT into PHC. The interviews began with the community-level, health-care providers, health system managers and decision makers, to conclude with stakeholders and experts in HAT and PHC. This sequence allowed to discuss the concerns of service users with those who organize and support the service.

**Table 1:** Number of FGDs and interviews by respondent categories and by study site

Categories	Yasa Bonga	Kongolo and Haut-Katanga	Bibanga	Kinshasa	Total
FGD					
Groups with community members	4	4	4		12
Interviews					
Community leaders	2	2	2		6
Care providers	2	2	2		6
Health system managers	2	2	1	3	8
Public health professionals	1	1	1	5	8
Researchers		2		2	4

FGD = focus group discussion.

### Data analysis.

The FGDs and the individual interviews were audio-recorded, transcribed literally and translated from the local language into French and transcribed by two independent translators. The first person translated from the local language into French and the second person back-translated the French version to the local language to check if the content remained intact during the translation. The translation of the manuscript into French to English was carried out by the services of a French language editor. All transcripts were supervised and verified by the principal investigator. The data were analyzed according to the thematic analysis approach.^21^ The data were coded independently by two researchers using the NVivo 11 software as analytical support. The two researchers identified a list of main themes and subthemes in an iterative process^22^ of reading and commenting. The themes were discussed with a third investigator until consensus was reached. Results were reported according to the consolidated criteria for qualitative research publications.^23^ Quotations from FGD discussions are identified by the code FGD followed by a code for the location and a two-digit number. Those from the interviews with health professionals are similarly identified as (Interview [INT] XX XX).

## RESULTS

Five main themes emerged from the data: the perception of the concept of integration; the utilization of health care; quality of care; financial barriers; and funding of HAT.

### Integration, a positively perceived concept.

In the discussions with health professionals, one central idea emerged that sleeping sickness or HAT must be seen as equal to other diseases such as malaria, tuberculosis and HIV/AIDS. A patient with HAT should be treated like any other sick person, and the quality of care should not depend on the pathogen. One of the health professionals interviewed in Kinshasa put it this way:

*“*[Integration] *is a good thing, an ill person, he is not a trypa patient, he is not a malaria patient, he is not an AIDS patient, he is not a tuberculosis patient, it is an ill person, an individual, whom the physician must take care of correctly and in all his pathologies.”* [INT KN PA 32].

Most care providers, managers, experts, and researchers moreover argued that integrating HAT services into general health facilities is advantageous for two main reasons, because it will lead to diagnosing more cases, and for reasons of efficiency.

But most of the professionals stated that integration into general health services is a way to reduce the cost of current HAT control activities and make it more sustainable. This is clearly said by one of the interviewed nurses,

“*The program puts all its money into mobile units. It costs too much, not only to the Congolese state but also to the partners who give the money. It’s expensive! ... If the health centers managed to take care of this disease, this would certainly solve some problems*.” [INT YB IT 18].

There were few dissenting voices on this overall positive perception evoked by managers who anticipated that integration would lead to loss of employment and staff benefits in the country program.

*“There is a fear of seeing a job disappearing because a job has some corollary benefits ...” *[INT MP KT 28].

So overall, the interviewed health professionals were largely in favor of integration. In the communities, on the other hand, opinions were more divided. Several people thought that HAT is a disease that requires a specialized service:

*“There is no rule that all the nurses at the center have to take care of sleeping sickness. Those who treat trypa have specifically studied for this. It’s not the business of all the nurses.”* [FGD YB 12].

Another focus group from the same area was even more explicit that general health services and sleeping sickness control should not be conflated:

“*We should not mix up Health and Fometro*” [FGD YB 9],

where “Health” refers to the (facilities of the) Ministry of Health and “Fometro” (Tropical Medical Fund) refers to the vertical program for the control of HAT (FOMETRO being the name of a nongovernmental organization that engaged in HAT control in the past). This claim was usually made in communities in areas of high endemicity such as that of Yasa Bonga, and the underlying reason was that they thought HAT screening and treatment was a very complex process that demanded special skills and could not be entrusted to a regular nurse.

Nonetheless, many focus groups welcomed the idea to have HAT diagnosis and care available year round at the nearby health center. They lamented about the current unavailability of HAT care and quoted the proximity of health centers as the main advantage of integration.

*“Suffering it is! If someone is suffering from this disease, one needs to travel a distance of 75 km from here until Kongolo; it is there that he will be tested and that he will be treated.”* [FGD KO 1].

In this sense, care providers and communities concurred to some extent that integration is a positive process: it will enhance access to care, increase the number of people screened, and in this way lead to beneficial impact. Although obviously the provision of HAT screening and treatment services in multipurpose health facilities de facto brings care closer to the communities, however, this proximity will not automatically lead to the use of the service, as discussed in the next theme.

### Health-seeking behavior.

Although sleeping sickness was seemingly well known by the community, yet this knowledge is fragmented. Symptoms of the advanced stage are usually recognized but the early, asymptomatic, phase of the disease was less well known, according to the professionals who claimed that at the early stage, people did not feel the need to go for screening. Early signs, if any, are often mistaken for malaria.

*“… the clinical picture of HAT corresponds to that of malaria. But in the mentality of our people, we can have malaria and stay in the village. So, thinking that it is malaria, whereas it is not malaria and it is HAT.”* [INT YB MZ 27].

Both health professionals and communities mentioned the existence of multiple care providers and multiple options along the path to seek care. The choice of provider depends on the perceived cause of the illness. For example, if a certain illness is seen as the result of a curse, people will in that case resort to traditional medicine or churches rather than to allopathic medicine.

*“If someone falls ill, he will become the victim of multiple slanders: he has ‘fetished,’ he is bewitched, or he has stolen something and was cast a bad spell”* [FGD YB 8];

*“… because the diseases are different: for some diseases, when we go to the health center, they do not offer any solution; when we go with the patient to our diviners, there they test them, they have the eyes.‡ Sometimes we go to prayer, to pray for him, and there, if it does not work, then we despair,…”* [FGD KO 3].

The health center is rarely used in first intention, according to the respondents. Often self-medication is used first and if there is no improvement, then only people would resort to a health facility. However, several times it was mentioned that financial barriers prevented many people from consulting health-care centers. Sociocultural barriers were evoked as well, as shown in the quotation as follows from the Katanga province.

*“Especially among our friends the bamukwetu ... these are the pygmies ... they are the ones who are there and who are “complexés †.” This kind of people is used to forests; they can go into the forest, and they may be affected by this disease, but they do not want to take the test to find out if they have the disease or not, they will stay over there.”* [FGD KO 1].

Several focus groups also pointed to gender bias, as it seems that men in the community more often than women think that attending HAT screening is a waste of time.

“*At screening, women are more numerous, most women respond, it is more men who do not respond in great numbers ... Men find it a waste of time. They are busy*.”[FGD YB 10].

Professionals and community, thus, evoked several barriers to effective HAT diagnosis and care, even if offered at close proximity and year round in health centers. As HAT itself is only symptomatic from the second stage, patients will not spontaneously seek care early. The care offered at health centers is not the first choice for communities, and several barriers exist; first and foremost of financial nature, but also sociocultural, including gender. Communities went even much further in their assessment of the care offered at the primary care level, and spontaneously brought up quality of care issues.

### Quality of care.

The communities explicitly stated that the quality of care offered by the first-level health services is not optimal. The frequent absenteeism of the nurses, their limited skills and the lack of equipment are quoted as compromising the quality of care. As salaries are insufficient, nurses have to engage in income-generating activities for their survival, such as farming.

*“There are problems; you arrive, you find the nurse who is going to treat the patient, then he goes to the farm so that he will see the patient again at 8 pm. If you go to the center at 7 am, you are not going to find this nurse. If the child had a life-threatening illness, he would die, these are the difficulties.”* [FGD KO 2].

Health-care providers pointed out that the integration of HAT screening and treatment would lead to an increase in their workload. Moreover, the free HAT screening service was seen as a financial shortfall for the health center and as a source of demotivation for care providers who felt they are poorly paid. This has to be understood in a context where primary care centers depend for their operations on out-of-pocket payments by the patients. These patient fees are used to pay an incentive to staff and some recurrent costs.

“*Free health care is a good thing for the patient. A destitute person can come and get treated free of charge, but on the side of the nurse, if he is not motivated he will not do his job properly, in the end he does not find his way*.” [INT KS IT 16].

Many professionals pointed out to the need for additional training.

“*We have to train the staff ... In the normal training course of the nurse or the* doctor, *we talk about trypanosomiasis, but we do not really go into depth, we have to train him, talk a little about what happens in practice*…” [INT MI 36].

Public health experts stressed that it is difficult to maintain the performance of trained people, especially in HAT diagnosis in the current low-prevalence context, where HAT cases are getting increasingly rare in endemic areas.

“*As the disease goes down, multi-purpose staff may not be competent and may not be able to diagnose and [they will] miss the disease*.” [INT PA 33].

Last but not the least, health facilities should be supplied with screening and diagnostic devices and medicines.

“*We cannot talk about integration if the drugs or the materials for detecting this disease are not available, so it’s really a set of several elements that we need to put together so that we can talk about integration into a basic health system*.” [INT KO MZ 25].

In summary, community and professionals alike stressed the importance of quality of HAT care. The current care package delivered by the specialized program is perceived as complex and of good quality, and one needs to maintain that quality level after integration. However, several attributes of the current health system jeopardize that quality of care (poor remuneration of health workers, insufficient training, lack of equipment, etc.).

### Financial barriers.

Opinions on financial issues related to HAT care diverged strongly between health professionals and community. Many health professionals stated that offering HAT care for free in the health center was not “coherent” with the Congolese health policy because health care is not free at the primary care centers. This was expressed by a district medical officer as follows:

“*If at the level of our country health care is not free, I do not see why some pathology must be treated free of charge! ... On the left, it says it’s free, and when they come to you, they say ‘but how is this possible, there it says free care and why not here ...’ There are other exams besides the trypanosomiasis tests that the patient has to take because we do not treat one illness, but the patient*.” [INT YB MZ 27].

Other arguments evoked against the provision of free HAT care were potential financial shortfalls to health facilities while increasing the volume of work, and doubts casted about the quality of service if it was offered for free.

“*It has always been said that what is scarce is expensive but something* that is *distributed free of charge, at times we wonder if it is really good quality or not! Because even when you go to the market, you will find that the things that cost less are generally poor quality*.” [INT YB IT 18].

There were only few professionals, mainly belonging to the group currently involved in HAT control, who evoked positive arguments about free HAT care, in terms of improved coverage. Contrastingly, communities emphasized repeatedly that having to pay for health care was a real barrier to accessing health centers because of their precarious social situation. Some people reported that even the HAT screening by mobile units is not always affordable as they sometimes have to pay for “a medical certificate” (a fee that was officially abandoned by the PNLTHA 9 years ago).

“*Yes, the 300 Francs Congolais discourage some people. This may be the major cause why people do not attend. The mobile team rejects people who do not have 300 Francs Congolais*.” [FGD YS 10].

For the community, free HAT care was an essential element that would promote access to care; this opinion was shared by only few professionals.

### Funding for HAT.

Only the group of health professionals elaborated further on the financing of health care in general and HAT in particular. They pointed out that “integrated health care” was not always perceived in the same way by funders and DRC policy makers. In their opinion, many international donors prefer to finance disease-specific programs rather than putting their resources in a common basket and seem more concerned about achieving their specific goals than strengthening the health system as a whole.

“*The partners are worried about funding for trypano spent in other domains ... it is maybe a kind of egoism—asking why our money is going to go to other activities, we will not know how to measure because they have their own indicators*.” [INT MP 28].

According to our interviewees, donors supporting only single-disease issues contribute to fragmenting the health system while health should be seen as a whole.

“*It would suffice to sit at the same table and to say that we are there to support the same system, a patient with trypano, can have malaria and therefore we must make the diagnosis of malaria, he can have renal insufficiency, we must make the diagnosis of renal insufficiency, so it is necessary to solve his problem, and therefore a patient remains a sick person, he must be considered as a whole*.” [INT MP 28].

In summary, donors were perceived as mainly interested in single-disease issues, with little interest in structurally supporting the health system. The health professionals we interviewed were of the opinion that this approach considerably weakened the system.

## DISCUSSION

The main finding of our study is that the concept of integrated HAT service delivery was welcomed in a positive way by communities in endemic areas and professionals belonging both to the general health services and the disease-control program divisions. The community particularly appreciated the proximity offered by fixed health facilities but feared possible financial barriers. Care providers, managers, experts, and researchers stressed the comprehensive nature of health care, and the potential gains in terms of coverage, cost reduction, and sustainability. Despite the fact that the management of HAT is complex, care providers and public health experts hardly objected to it being integrated in first-line health services. Acknowledging it will increase their workload, our respondents also showed a certain enthusiasm to take on new responsibilities. A similar observation was made by Arun et al.,^24^ who noted the satisfaction of caregivers with their new role in the integrated provision of mental health care. Arun et al.^24^ stressed that vertical programs have advantages over integrated approaches in certain circumstances. In a context of disease elimination, vertical programs have the advantage of being more focused, faster, and more goal-oriented. In a context of low prevalence, they are more likely to remain specialized and competent (R. A. Atun, S. Bennett and A. Duran, unpublished data). Criel and De Brouwere^25^ also state that a situation of low prevalence may constitute an argument for not integrating because, in the absence of cases, it is often difficult for staff to maintain the skills.

All categories of interviewees in our study placed great emphasis on the need for training of health professionals and on the need to supply health centers with commodities and equipment for HAT screening and diagnosis. The training of primary care staff is needed to optimize the quality of the integrated HAT services. Mitashi et al.^10^ described how the lack of trained personnel was a major obstacle to carrying out HAT control activities in multi-purpose services. Workneh et al.,^26^ studying the integration of diabetes and tuberculosis management also identified the lack of knowledge about diabetes as an obstacle to the delivery of integrated services. Such training must be continuous, as in the long term and in a low-prevalence context, it will be difficult to maintain technical skills if providers do not see enough HAT cases. In addition, multipurpose health-care staff may not be particularly motivated to take part in training courses about a rare health problem.^25^

In addition to the challenge of continuing education, care structures must be provided with the necessary equipment to ensure quality care because integrating activities into a nonfunctional system is of limited value.^27^ The lack of equipment characterizing the majority of DRC’s health-care facilities and the enormous challenge of training could have a negative impact on the quality and availability of HAT care. Some health-care providers fear an increased workload without financial compensation, which would have a negative impact on motivation. The community may then lose confidence in their capacity, resulting in an even lower rate of community use of services (A. Mpanya, unpublished data). The study by Meda et al.^28^ in Burkina Faso showed that implementation of a decentralized approach to tuberculosis prevention in rural areas was effective under certain conditions, including a functioning health district system. This is consistent with the opinions of some health professionals in our study who insisted on the operationality of the health-care service as the essential prerequisite for integration.

Authors such as Criel et al.^29^ emphasize that integration offers an opportunity to extend coverage of disease control program activities, which is an important dimension in the current elimination context. However, to have a positive impact on HAT screening coverage in the current context, it is important to avoid financial barriers in integrated approaches. Our study clearly highlighted the use of other therapeutic itineraries by the community, motivated by the unaffordability of the health care at the centers. Respondents agreed that free services are essential to maintain access to screening and treatment of HAT. A qualitative study by Robays et al.^30^ had clearly demonstrated that a minor fee asked by the program was an obstacle for participating in the screening, which had motivated the PNLTHA to make it free thereafter. These results are similar to those of our study, which showed that even an amount as low as 300 Francs Congolais was a real barrier for accessing HAT care. Screening by mobile teams should now be free, and this is made possible thanks to the support by donors (PNLTHA-DRC, unpublished data). Important financial barriers in integrated care have also been mentioned by Workneh et al.^26^ In the current context, paid integrated screening will not be a valid substitute for the vertical program. As long as HAT care is not free, part of the population and probably those most at risk will resort to alternative therapeutic itineraries and will not to get tested for HAT via the health centers. Therefore, as it is essential to ensure coverage of those most in need of HAT screening; financial barriers within the primary care system need to be addressed. In addition to needing to meet the needs of HAT infected persons, the country will not be able to achieve its elimination targets until all who are infected by HAT have been treated.

Two limitations of this study should be mentioned. First, for budgetary and time constraints, we could not include any staff of mobile units, which leaves the picture of all stakeholders somewhat incomplete. Second, some interviews with provincial managers and researchers may have been influenced by the fact that the principal investigator who conducted them was a colleague (former manager and researcher) at some point in his career.

## CONCLUSION

In conclusion, although integration of HAT screening and treatment is welcomed by the respondents in this study, they highlighted several implementation challenges. First, to achieve the required quality of care, health centers should be fully equipped and operational. Caregivers should be well-trained as current HAT case management is complex, although this may become simpler in the future with novel treatment regimens. Recent efforts to improve HAT screening in Uganda’s routine health-care activities described by Wamboga et al.^31^ can help to overcome some of the implementation challenges. In this country, the improved algorithm for passive screening and confirmatory diagnosis of HAT has been an indispensable tool, where the public health infrastructure is underutilized. To ensure coverage of those most in need of HAT screening, financial barriers within the primary care system need to be addressed.

## Footnotes

† Word in French in the original kiswahili text. Used here in the meaning of “embarrassed.”

‡ “They have the eyes” in the meaning of “They see the cause of the disease by divination.”

## References

[1] Brun R Blum J Chappuis F Burri C, 2010 Human African trypanosomiasis. Lancet 375: 148–159. 19833383 10.1016/S0140-6736(09)60829-1

[2] Franco JR Simarro PP Diarra A Jannin JG, 2014 Epidemiology of human African trypanosomiasis. Clin Epidemiol 6: 257–275. 25125985 10.2147/CLEP.S39728 PMC4130665

[3] WHO, 2013 Control and surveillance of human African trypanosomiasis. World Health Organ Tech Rep Ser 2013: 1–237. 24552089

[4] Buscher P Cecchi G Jamonneau V Priotto G, 2017 Human African trypanosomiasis. Lancet 390: 2397–2409. 28673422 10.1016/S0140-6736(17)31510-6

[5] Blum J Schmid C Burri C, 2006 Clinical aspects of 2541 patients with second stage human African trypanosomiasis. Acta Trop 97: 55–64. 16157286 10.1016/j.actatropica.2005.08.001

[6] Buguet A Bourdon L Bisser S Chapotot F Radomski M Dumas M, 2001 Sleeping sickness: major disorders of circadian rhythm [article in French]. Med Trop (Mars) 61: 328–339. 11803823

[7] Kennedy PG, 2006 Human African trypanosomiasis-neurological aspects. J Neurol 253: 411–416. 16541214 10.1007/s00415-006-0093-3

[8] Simarro PP Diarra A Ruiz Postigo JA Franco JR Jannin JG, 2011 The human African trypanosomiasis control and surveillance programme of the World Health Organization 2000–2009: the way forward. PLoS Negl Trop Dis 5: e1007. 21364972 10.1371/journal.pntd.0001007 PMC3042999

[9] Eperon G Balasegaram M Potet J Mowbray C Valverde O Chappuis F, 2014 Treatment options for second-stage gambiense human African trypanosomiasis. Expert Rev Anti Infect Ther 12: 1407–1417. 25204360 10.1586/14787210.2014.959496 PMC4743611

[10] Mitashi P Hasker E Mbo F Van Geertruyden J Kaswa M Lumbala C Boelaert M Lutumba P, 2015 Integration of diagnosis and treatment of sleeping sickness in primary healthcare facilities in the Democratic Republic of the Congo. Trop Med Int Health 20: 98–105. 25329353 10.1111/tmi.12404

[11] Simarro PP Sima FO Mir M Mateo MJ Roche J, 1991 Control of human African trypanosomiasis in Luba in equatorial Guinea: evaluation of three methods. Bull World Health Organ 69: 451–457. 1934239 PMC2393243

[12] WHO, 1998 Control and surveillance of African trypanosomiasis. World Health Organ Tech Rep Ser 881: 1–114. 10070249

[13] Holmes P, 2014 First WHO meeting of stakeholders on elimination of gambiense human African trypanosomiasis. PLoS Negl Trop Dis 8: e3244. 25340404 10.1371/journal.pntd.0003244 PMC4207655

[14] Simarro PP Cecchi G Franco JR Paone M Diarra A Priotto G Mattioli RC Jannin JG, 2015 Monitoring the progress towards the elimination of gambiense human African trypanosomiasis. PLoS Negl Trop Dis 9: e0003785. 26056823 10.1371/journal.pntd.0003785 PMC4461311

[15] Pepin J Guern C Milord F Bokelo M, 1989 Integration of African human trypanosomiasis control in a network of multipurpose health centers. Bull World Health Organ 67: 301–308. 2766452 PMC2491256

[16] Laveissiere C Meda AH Doua F Sane B, 1998 Detecting sleeping sickness: comparative efficacy of mobile teams and community health workers. Bull World Health Organ 76: 559–564. 10191551 PMC2312482

[17] Franco JR Simarro PP Diarra A Ruiz-Postigo JA Jannin JG, 2014 The journey towards elimination of gambiense human African trypanosomiasis: not far, nor easy. Parasitology 141: 748–760. 24709291 10.1017/S0031182013002102

[18] Lumbala C 2015 Human African trypanosomiasis in the Democratic Republic of the Congo: disease distribution and risk. Int J Health Geogr 14: 1–14. 26047813 10.1186/s12942-015-0013-9 PMC4501122

[19] Van Nieuwenhove S Betu-Ku-Mesu V Diabakana P Declercq J Bilenge C, 2001 Sleeping sickness resurgence in the DRC: the past decade. Trop Med Int Health 6: 335–341. 11348528 10.1046/j.1365-3156.2001.00731.x

[20] Ochieng N Wilson K Derrick C Mukherjee N, 2018 The use of focus group discussion methodology: insights from two decades of application in conservation. Methods Ecol Evol 9: 20–32.

[21] Kohn L Christiaens W, 2014 Les méthodes de recherches qualitatives dans la recherche en soins de santé: apports et croyances. Reflets et Perspectives de la vie Économique LIII: 67–82.

[22] Mukamurera J Lacourse F Couturier Y, 2006 Des avancées en analyse qualitative: pour une transparence et une systématisation des pratiques. Recherches qualitatives 26: 110–138.

[23] Tong A Sainsbury P Craig J, 2007 Consolidated criteria for reporting qualitative research (COREQ): a 32-item checklist for interviews and focus groups. Int J Qual Health Care 19: 349–357. 17872937 10.1093/intqhc/mzm042

[24] Arun NN Mohan KI Parthasarathy R Karur BV, 1994 The perception and experience of health personnel about the integration of mental health in general health services. Indian J Psychiat 36: 18–21. PMC2972448 21743660

[25] Criel B De Brouwere V, 1997 Conditions, limites et potentiel de l’intégration. Van Lerberghe W Béthune X, eds. Intégration et Recherche. Antwerpen, Belgium: Studies in Health Services Organisation & Policy, 95–123.

[26] Workneh MH Bjune GA Yimer SA, 2016 Assessment of health system challenges and opportunities for possible integration of diabetes mellitus and tuberculosis services in south-eastern Amhara Region, Ethiopia: a qualitative study. BMC Health Serv Res 16: 135. 27095028 10.1186/s12913-016-1378-6 PMC4837556

[27] Roos B Van Brakel W, 1994 Integration of vertical projects into the basic health services: an example from the leprosy control project. Int Nepalese Med Assoc 32: 273–286.

[28] Méda ZC Huang CC Sombié I Konaté L Somda PK Djibougou AD Sanou M, 2014 Tuberculosis in developing countries: conditions for successful use of a decentralized approach in a rural health district. Pan Afr Med J 17: 198. 25396024 10.11604/pamj.2014.17.198.3094 PMC4228989

[29] Criel B Kegels G Van der Stuyft P, 2004 A framework for analysing the relationship between disease control programmes and basic health care. Trop Med Int Health 9: A1–A4. 10.1111/j.1365-3156.2004.01257.x 15189467

[30] Robays J Lefèvre P Lutumba P Lubanza S Kande V Van der Stuyft P Boelaert M, 2007 Drug toxicity and cost as barriers to community participation in HAT control in the Democratic Republic of Congo. Trop Med Int Health 12: 290–298. 17300638 10.1111/j.1365-3156.2006.01768.x

[31] Wamboga C Matovu E Bessell P Picado A Biéler S Ndung’u J Arez A, 2017 Enhanced passive screening and diagnosis for gambiense human African trypanosomiasis in north-western Uganda moving towards elimination. PLoS One 12: e0186429. 29023573 10.1371/journal.pone.0186429 PMC5638538

